# Return to Work, Demographic Predictors, and Symptomatic Analysis Among Healthcare Workers Presenting for COVID-19 Testing: A Retrospective Cohort From a United States Academic Occupational Medicine Clinic

**DOI:** 10.7759/cureus.19944

**Published:** 2021-11-27

**Authors:** Zaira S Chaudhry, Leslie Cadet, Akbar Sharip

**Affiliations:** 1 Occupational Medicine, Loma Linda University Medical Center, San Bernardino, USA

**Keywords:** return to work, occupational health, employees, healthcare workers, covid-19, sars-cov-2

## Abstract

Introduction

We sought to determine time to return to work (RTW) among healthcare workers (HCWs) with mild/moderate coronavirus disease 2019 (COVID-19) and identify predictors of COVID-19 test positivity and illness duration.

Methods

A retrospective review of HCWs presenting for COVID-19 testing/evaluation in December 2020 was performed to examine demographics, clinical characteristics, and RTW.

Results

Of 250 exposure incidents, 107 employees (42.80%) tested positive for severe acute respiratory syndrome coronavirus 2 (SARS-CoV-2). No significant differences between COVID-19 positive and negative HCWs were noted in terms of key demographics, including age, gender, and CDC risk scores. Cough (77.57% vs 56.64%, p = 0.001), fatigue (66.36% vs 51.05%, p = 0.015), fever/chills (65.42% vs 37.06%, p < 0.001), myalgia (57.01% vs 35.66%, p = 0.008), and change in smell/taste (38.32% vs 13.29%, p < 0.001) were more prevalent among COVID-19 positive versus negative HCWs. Change in smell/taste (p < 0.001, OR 3.592), cough (p = 0.001, OR 2.966), and fever/chills (p = 0.019, OR 2.107) were independently associated with COVID-19 test positivity. Mean time to RTW from symptom onset was 13.09 days for COVID-19 positive HCWs. Female gender (p = 0.020, + 3.20 days), older age (p = 0.014, + 2.22 days), and myalgia (p = 0.021, + 2.23 days) were predictive of longer illness duration.

Conclusion

Change in taste/smell, cough, and fever/chills were independently associated with COVID-19 test positivity. Among HCWs with mild/moderate COVID-19 infection, the mean time to RTW was approximately 13 days with female gender, older age, and myalgia being predictive of delayed RTW.

## Introduction

According to the World Health Organization (WHO), there have been an estimated 252 million cases of coronavirus disease 2019 (COVID-19) reported globally as of mid-November 2021 [1]. Since the pandemic began in early 2020, workplace absenteeism due to illness has increased substantially, especially among workers in essential critical infrastructure occupations across various industries (e.g. healthcare, emergency response, grocery/food services, agriculture, etc.) [2]. In fact, illness-related work absenteeism has reached record highs in the United States (US) during the COVID-19 pandemic [3]. Healthcare workers (HCWs), in particular, have an increased risk of being exposed to the severe acute respiratory syndrome coronavirus 2 (SARS-CoV-2) virus and acquiring COVID-19 infection [4]. The observed increased risk in this occupational group has been attributed to prolonged exposure to known infected patients, exposure to patients with unrecognized COVID-19 infection, and inadequate access to or improper use of personal protective equipment [5-6]. While vaccination efforts are underway in many countries, several challenges in ensuring global and equitable access to immunization remain along with growing concerns regarding vaccine hesitancy, both of which present a formidable challenge to attaining herd immunity on a global scale [7-8]. Despite early vaccination efforts, there is still a significant segment of the global workforce that remains susceptible to developing COVID-19, especially workers with public-facing occupations. 

Given that this rampant disease continues to have the potential to impact a significant proportion of essential working populations who may be at greater risk of being exposed to the SARS-CoV-2 virus, it is imperative to examine associations between patient demographics, symptomatology, test positivity, and illness duration among employees presenting for COVID-19 testing following exposure. Clinicians certainly stand to benefit from understanding the predictive value of different viral symptoms, which may help guide clinical decision-making. Moreover, determining whether certain demographics are associated with longer illness duration or time to return to work among a working population may also provide valuable insights regarding the broader economic and societal impact of COVID-19. To date, several studies exploring COVID-19 symptomatology and test positivity among HCWs in various geographic regions have been published with variable findings [9-14].

The present retrospective cohort study of clinical and non-clinical HCWs exposed to SARS-CoV-2 who presented for evaluation at our academic medical center’s Occupational Medicine Clinic (OMC) sought to: 1) examine the frequency of viral symptoms and their predictive value in diagnosing COVID-19; 2) examine the association between employee demographics and SARS-CoV-2 test positivity; 3) determine the mean number of days from symptom onset to discharge from the clinic (return to work); 4) examine the association between employee demographics and illness duration, and 5) examine the association between symptoms and illness duration.

This article was previously presented as a meeting poster and podium presentation at the 2021 Western Occupational Health Conference (WOHC) in Phoenix, Arizona on October 2, 2021.

## Materials and methods

Data collection

After obtaining approval from the Loma Linda University Institutional Review Board (IRB Approval #5210060), a retrospective chart review of employees at our academic medical center in Southern California was performed in February 2021. A waiver of informed consent was obtained given the retrospective nature of the study. The institution’s electronic health record (EHR) system was queried to generate a list of all patients who underwent COVID-19 testing ordered by a physician at our institution’s OMC between December 1, 2020, and December 31, 2020; this particular time period was selected because there was a surge of COVID-19 cases in our geographic region during December 2020. The inclusion criteria for this study were as follows: employees over the age of 18 who underwent COVID-19 testing between December 1, 2020, and December 31, 2020, after presenting to the OMC with concern for COVID-19 infection following exposure to SARS-CoV-2. Employees who underwent COVID-19 testing without being evaluated at our OMC (e.g. clinical evaluation by their primary care provider or another external provider, testing without clinical evaluation) were excluded. It is important to emphasize that this cohort represents a relatively young and healthy population with mild to moderate disease as severe cases were excluded given that such cases warrant a higher level of care than could be provided at an outpatient occupational medicine clinic. Therefore, the study population consisted of all HCWs employed in clinical and non-clinical roles at our institution who presented to the OMC for SARS-CoV-2 testing and clinical evaluation via telehealth modalities (e.g. video, telephone) during the study period.

In the period between December 1, 2020, and December 31, 2020, a total of 246 employees were tested and evaluated for suspected COVID-19 following exposure to the virus. Four of these employees presented to the clinic twice during the month of December 2020 for separate exposure incidents, which resulted in a total of 250 exposure incidents evaluated at our clinic during the study period. 

Demographic data recorded included age, gender, race, and occupation. A previously validated COVID-19 disease risk score derived from guidance set forth by the Centers for Disease Control and Prevention (CDC) was used as a proxy for comorbidities [15-16]. This score indicates the number of risk factors a patient over the age of 18 has for hospitalization and in-hospital mortality if they develop a COVID-19 infection. In accordance with CDC guidance, it takes into account both age and sex as well as the following risk factors: congestive heart failure, coronary artery disease, end-stage renal disease, end-stage liver disease, chronic pulmonary disease, hypertension, diabetes mellitus, obesity, immunocompromised states, pregnancy status, and nursing home residence [15-16]. Risk scores range from 0 (lowest risk) to 15 (highest risk) with scores divided into the following three risk categories: green (score 0-2), yellow (score 3-5), and red (score 6-15) [15]. This risk score is automatically calculated and reported in the EHR system utilized by our institution (Epic EHR system, Epic Systems Corp, Verona, Wincosnin).

Per our state’s public health department, an exposure was defined as being in close contact (within 6 feet or less for a total of 15 minutes or more) with a COVID-19 infected individual. If known, the suspected date of exposure and symptom onset were recorded. The presence or absence of the following symptoms were recorded: fever/chills, fatigue/malaise, myalgia, cough, shortness of breath (SOB), nasal congestion, sore throat, diarrhea, nausea, and loss of smell/taste (anosmia/ageusia). The timing of SARS-CoV-2 nasopharyngeal reverse transcription polymerase chain reaction (RT-PCR) testing, number of tests performed, and number of positive tests were recorded. RT-PCR tests were performed by various laboratories, including our own institution’s laboratory, and different testing sites depending on where the employee opted to undergo testing; regardless, all available test results were reviewed and confirmed as either positive or negative by one of our providers. COVID-19 cases were defined as having at least one positive nasopharyngeal RT-PCR during the study period; serology and rapid testing alone were not considered diagnostic and were followed by confirmatory RT-PCR testing. Either a single or multiple RT-PCR test strategy was utilized depending on provider preference and clinical suspicion for COVID-19. Outcome data recorded consisted of ER visits, ICU admissions, mortality, illness duration, and discharge disposition. Of note, vaccination status was not readily available during our data collection as COVID-19 vaccines first became available to our healthcare staff in mid-December 2020; however, it can be assumed that this sample represents a largely unvaccinated to partially vaccinated group of healthcare workers. 

Illness duration was defined as the number of days from symptom onset to discharge from clinic with clearance to return to regular duty without work restrictions. Discharge disposition was defined as the patient’s self-reported clinical status at the time of discharge from our clinic, which was classified as follows: symptom resolution, symptomatic improvement, no change in symptoms, worsening of symptoms, or unknown (loss to follow-up). CDC guidance was used by all providers at the OMC to determine minimum duration of self-isolation for HCWs: at least 10 days from symptom onset for mild to moderate disease in non-immunocompromised patients with symptomatic improvement and absence of fever for at least 24 hours without the use of antipyretics [17]. 

Statistical methods

The de-identified dataset was analyzed using quantitative methodologies with commercially available statistical software (SAS® University Edition; SAS Institute, Cary, North Carolina). The association between demographics, CDC risk score, clinical symptomatology variables, and COVID-19 positivity were tested using descriptive/univariable and multivariable analyses. For descriptive statistics, employees were compared by COVID-19 positivity using means and Chi-squared tests with two-sided alphas for all hypotheses. Non-parametric tests were used as a test of sensitivity of assumptions of the distributions. For univariable and multivariable associations with the positivity outcome, logistic regression analyses were used. Variables were added to test hypotheses of demographic and CDC risk score associations as a priori hypotheses, and the same methods were used to examine clinical symptomatology as main effect exposure variables. Model assumptions were tested using log likelihood ratio tests of indicator variables. A linear regression model was used to examine the association between illness duration and demographics, CDC risk score, and clinical symptomatology variables. Tests of normality revealed a positive skew of the illness duration data. To account for the non-normality of the illness duration data, the outcome was log transformed. Final results are given as a back transformation. Cases with missing data points were excluded from the analyses on a variable-by-variable basis. An a priori alpha level of 0.05 was set as the cutoff to determine statistical significance for all statistical analyses performed in this study.

## Results

Sample demographics

Of the 250 exposure incidents evaluated during the study period, 107 cases (42.80%) were positive and the remaining 143 cases (57.20%) were negative for SARS-CoV-2 based on RT-PCR testing results. A comparison of key demographics between both groups is presented in Table I. The mean age among COVID-19 positive and negative employees was 37.24 years and 36.99 years, respectively (p = 0.54). The COVID-19 positive group was 72.90% female and 27.10% male, whereas the COVID-19 negative group was 67.83% female and 32.17% male (p = 0.390). The majority of patients (91.60%) identified as White, Hispanic, or Asian as reported in Table 1. Moreover, mean CDC COVID-19 risk scores were 0.92 and 0.99 among COVID-19 positive and negative employees, respectively (p = 0.854).

**Table 1:** Demographics of employees presenting for COVID-19 testing and clinical evaluation according to test positivity. Covid 19: coronavirus disease 2019; n: number; N: number; SD: standard deviation; SARS-CoV-2: severe acute respiratory syndrome coronavirus 2; PCR: polymerase chain reaction

SARS-CoV-2 PCR Result	Negative (n = 143)		Positive (n = 107)		
	Mean/N	SD/%	Mean/N	SD/%	P-value
Age	36.99	10.55	37.24	11.42	0.594
Sex					0.390
Female	97	67.83	78	72.90	
Male	46	32.17	29	27.10	
Race					0.232
White	49	34.27	44	41.12	
Hispanic	40	27.97	32	29.91	
Black	4	2.8	6	5.61	
Asian	44	30.77	20	18.69	
Others	1	0.7	1	0.93	
CDC risk score	0.99	1.14	0.92	0.93	0.854
Occupational group					0.116
Clinical	115	80.42	94	87.85	
Non-Clinical	28	19.58	13	12.15	

Occupational roles 

**Figure 1:**
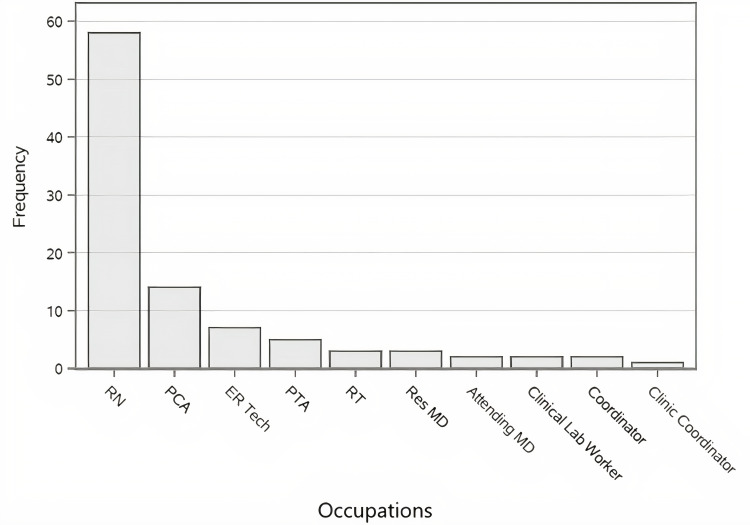
Top 10 occupations among employees with symptomatic COVID-19. COVID-19: coronavirus 2019; RN: registered nurse; PCA: patient care assistant; ER tech: emergency room technician; PTA: physical therapy assistant; RT: respiratory therapist; Res MD: resident physician; attending MD: attending physician

In the COVID-19 negative group, 80.42% of employees were employed in clinical roles providing direct patient care, and 19.58% were employed in non-clinical roles. Likewise, 87.85% of employees in the COVID-19 positive group were employed in clinical roles providing direct patient care, and 12.15% were employed in non-clinical roles. However, this observed difference in clinical versus non-clinical roles between groups was not statistically significant (p = 0.116). It is important to note that the majority of patients with confirmed symptomatic COVID-19 infection in our cohort were registered nurses. The top ten occupations among patients with symptomatic COVID-19 are presented in Figure 1.

Symptomatic analysis

Table 2 summarizes the symptomatic analysis of employees presenting for COVID-19 testing and clinical evaluation according to test positivity. COVID-19 positive employees were more likely to report experiencing cough (77.57% vs 56.64%, p = 0.001), fatigue (66.36% vs 51.05%, p = 0.015), fever/chills (65.42% vs 37.06%, p < 0.001), myalgia (57.01% vs 35.66%, p = 0.008), and change in smell/taste (38.32% vs 13.29%, p < 0.001) than their negative counterparts. No statistically significant group differences were noted in terms of the prevalence of SOB, sore throat, nasal congestion, nausea/vomiting, or diarrhea (p > 0.05). 

**Table 2:** Symptomatic analysis of employees presenting for COVID-19 testing and clinical evaluation according to test positivity. Covid 19: coronavirus disease 2019; n: number; N: number; SARS-CoV-2: severe acute respiratory syndrome coronavirus 2; PCR: polymerase chain reaction

SARS-CoV-2 PCR Result	Negative (n = 143)		Positive (n = 107)		
	N	%	N	%	P-value
Fever or chills					<0.001
No	90	62.94	37	34.58	
Yes	53	37.06	70	65.42	
Cough					0.001
No	62	43.36	24	22.43	
Yes	81	56.64	83	77.57	
Shortness of breath					0.354
No	109	76.22	76	71.03	
Yes	34	23.78	31	28.97	
Fatigue					0.015
No	70	48.95	36	33.64	
Yes	73	51.05	71	66.36	
Myalgia					0.008
No	92	64.34	46	42.99	
Yes	51	35.66	61	57.01	
Sore throat					0.621
No	61	42.66	49	45.79	
Yes	82	57.34	58	54.21	
Nasal congestion					0.226
No	105	73.43	71	66.36	
Yes	38	26.57	36	33.64	
Nausea or vomiting					0.278
No	127	88.81	90	84.11	
Yes	16	11.19	17	15.89	
Diarrhea					0.695
No	119	83.22	87	81.31	
Yes	24	16.78	20	18.69	
Change in smell or taste					<0.001
No	124	86.71	66	61.68	
Yes	19	13.29	41	38.32	

Outcomes and illness duration analysis

Seven (6.54%) COVID-19 infected patients and three (2.10%) COVID-19 negative patients presented to the ER during the study interval. Although there were 2.3 times more ER visits among COVID-19 infected patients than their COVID-19 negative counterparts, this difference was not statistically significant (p = 0.077). No ICU admissions were observed in either group. Moreover, no patient deaths occurred in either group during the study period. Of note, 29 (27.10%) COVID-19 positive patients and 14 (9.79%) of COVID-19 negative patients reported a similar illness among their household members, and this difference was statistically significant (p < 0.001).

Of the 250 exposure incidents, 103 (41.20%) were industrial (work-related), 145 (58.00%) were non-industrial (non-work related), and 2 (0.80%) were regarded as undetermined based on the employee's self-reported exposure history. Five (4.67%) COVID-19 infected patients and 11 (7.69%) COVID-19 negative patients were lost to follow-up. As of January 31, 2021, three (2.80%) COVID-19 infected patients remained under the care of our clinic due to residual symptoms. The remaining patients in both groups were discharged during the study period.

Among COVID-19 infected patients, on average, there were 4.58 days (3.44, 5.72) between the COVID-19 exposure date and symptom onset (incubation period), 8.38 days (7.01, 9.75) between the exposure date and the initial visit to our clinic, 9.32 days (8.13, 10.53) between the initial visit and the final visit (discharge), and 13.09 days (11.83, 14.35) between the date of symptom onset and the final clinic visit (discharge). Among COVID-19 negative patients, on average, there were 4.16 days (3.76, 4.61) between the initial visit and the final visit (discharge) and 7.12 days (6.45, 7.80) between the date of symptom onset and the final clinic visit (discharge). As expected, COVID-19 negative patients had a much shorter interval from symptom onset to discharge (p < 0.01) and from the initial visit to discharge (p < 0.01), and therefore returned to work much sooner than their COVID-19 positive counterparts. On the day of discharge from clinic, COVID-19 infected patients subjectively reported symptomatic improvement (77.78%), complete resolution of symptoms (17.17%), no change in symptoms (2.02%), or worsening of symptoms (0.93%).

Logistic regression modeling of demographic and clinical predictors of COVID-19 test positivity and illness duration

**Figure 2:**
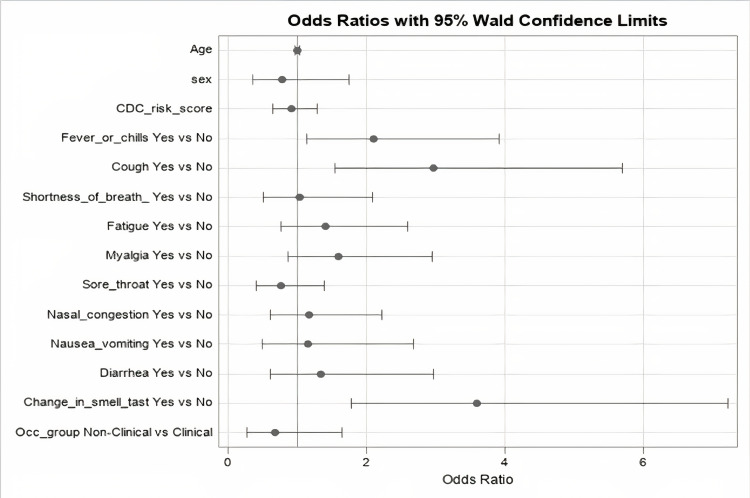
Demographic and clinical predictors of COVID-19 positivity. COVID-19: coronavirus disease 2019; Occ: occupational

Between group differences were explored in univariable and multivariable logistic regression models with a binary variable for COVID-19 positivity as the outcome as summarized in Table 3 and Table 4. The multivariable model confirmed that age (p = 0.936, OR 0.999, 95% CI 0.973-1.025), gender (p = 0.229, OR 0.657, 95% CI 0.331-1.303), CDC risk score (p = 0.821, OR 0.966, 95% CI 0.718-1.300), and working in a non-clinical versus clinical role (p = 0.396, OR 0.680, 95% CI 0.279-1.655) were not significant predictors of COVID-19 test positivity. A marginally significant association indicating a two-fold lower odds of test positivity was noted among Asian vs White patients (p = 0.052, OR 0.513, 95% CI 0.262-1.004); however, race was otherwise not noted to be predictive of test positivity. In a multivariable model of presenting symptoms, the presence of change in smell/taste (p < 0.001, OR 3.592, 95% CI 1.787-7.218), cough (p = 0.001, OR 2.966, 95% CI 1.544-5.695), and fever/chills (p = 0.019, OR 2.107, 95% CI 1.133-3.919) were independently associated with COVID-19 test positivity as illustrated in Figure 2. 

**Table 3:** Demographic predictors of COVID-19 positivity. COVID-19: coronavirus disease 2019; M: male; F: female

Effect	Unit	Odds Ratio	95% Confidence Interval		P-value
Age	1	0.999	0.973	1.025	0.936
Gender M vs F	1	0.657	0.331	1.303	0.229
CDC risk score	1	0.966	0.718	1.300	0.821
Race Asian vs White	1	0.513	0.262	1.004	0.052
Race Black vs White	1	1.986	0.509	7.748	0.323
Race Hispanic vs White	1	0.895	0.48	1.669	0.728
Race Others vs White	1	1.003	0.061	16.617	0.998
Non-Clinical vs Clinical Occupation	1	0.680	0.279	1.655	0.396

**Table 4:** Multivariable model of demographic and clinical predictors of COVID-19 positivity. COVID-19: coronavirus disease 2019; M: male; F: female; Occ: occupational

Effect	Unit	Odds Ratio	95% Confidence Interval		P-value
Age (increase every 1-year)	1	1.000	0.971	1.03	0.997
Sex (M vs F)	1	0.792	0.358	1.75	0.564
CDC risk score (increase every 1 unit)	1	0.913	0.643	1.296	0.610
Occ group (Non-Clinical vs Clinical)	1	0.68	0.279	1.655	0.396
Fever or chills (Yes vs No)	1	2.107	1.133	3.919	0.019
Cough (Yes vs No)	1	2.966	1.544	5.695	0.001
Shortness of breath (Yes vs No)	1	1.031	0.509	2.09	0.932
Fatigue (Yes vs No)	1	1.416	0.774	2.593	0.259
Myalgia (Yes vs No)	1	1.604	0.873	2.945	0.128
Sore throat (Yes vs No)	1	0.762	0.416	1.394	0.378
Nasal congestion (Yes vs No)	1	1.169	0.615	2.219	0.634
Nausea or vomiting (Yes vs No)	1	1.157	0.499	2.679	0.734
Diarrhea (Yes vs No)	1	1.351	0.614	2.973	0.455
Change in smell or taste (Yes vs No)	1	3.592	1.787	7.218	<0.001

In terms of demographic and clinical predictors of illness duration among COVID-19 positive employees, female gender (p = 0.020, + 3.20 days), older age (p = 0.014, + 2.22 days), and the presence of myalgia (p = 0.021, + 2.23 days) were associated with a longer duration of illness. Race, CDC risk score, and the presence of other presenting symptoms were not noted to be predictive of illness duration among COVID-19 positive employees (p > 0.05). Table 5 summarizes demographic and clinical predictors of illness duration in days among employees who tested positive for COVID-19.

**Table 5:** Predictors of illness duration (days) among employees who tested positive for COVID-19. COVID-19: coronavirus 2019; Y: yes; N: no; M: male; F: female; Q1: first quartile; Q3: third quartile

Variable	Difference in illness days	P-value
CDC risk score (Q3 vs Q1)	0.05	0.942
Age (Q3 vs Q1)	2.22	0.014
Sex (M vs F)	-3.20	0.020
Hispanic vs White	1.41	0.239
Black vs White	0.59	0.804
Asian vs White	1.75	0.207
Others vs White	-1.22	0.789
Fever or chills (Y/N)	0.40	0.706
Cough (Y/N)	2.03	0.100
Shortness of breath (Y/N)	0.74	0.532
Fatigue (Y/N)	-0.08	0.937
Myalgia (Y/N)	2.23	0.021
Sore throat (Y/N)	-0.27	0.788
Nasal congestion (Y/N)	0.53	0.632
Nausea or vomiting (Y/N)	-1.18	0.368
Diarrhea (Y/N)	-0.90	0.512
Change in smell or taste (Y/N)	-0.84	0.419

## Discussion

The present study provides valuable insights regarding COVID-19 symptomatology and RTW in a large cohort of HCWs. In our cohort of employees presenting for SARS-CoV-2 testing and clinical evaluation in December 2020, 42.80% of patients had COVID-19 infection. We did not observe any statistically significant differences between COVID-19 positive and negative patients in terms of key demographics, including age, gender, and CDC risk scores. However, a marginally significant association indicating a two-fold lower odds of test positivity was noted among Asian vs White patients. In our cohort of HCWs, cough, fever/chills, fatigue, myalgia, and change in taste/smell were significantly more prevalent among COVID-19 positive versus COVID-19 negative patients. Moreover, in a multivariable model of presenting symptoms, the presence of a change in smell/taste, cough, and fever/chills were independently associated with COVID-19 test positivity. Mean illness duration or time to RTW from symptom onset was approximately 13 days for employees with confirmed COVID-19 infection, and female gender, older age, and the presence of myalgia were noted to be predictive of longer illness duration. While some of our results parallel previous findings of studies exploring COVID-19 symptomatology among HCWs, others appear to contradict prior findings. 

Similar symptom profiles have been reported among HCWs with confirmed COVID-19 infection in prior studies. For example, Fan-Yun et al. [9] conducted a retrospective study of HCWs in Massachusetts who underwent COVID-19 screening and testing from March to April 2020. Of the 592 HCWs tested, 83% had an initial positive SARS-CoV-2 assay. The multivariate-adjusted odds of a positive assay were increased with reports of three or more symptoms. The OR was increased with reports of fever and a temperature ≥ 37.5℃ (OR 3.49), myalgia (OR 1.83), and anosmia/ageusia (OR 7.21) [9]. Malenfant et al. [10] also performed a symptom analysis of HCWs tested for SARS-CoV-2 at their large California academic medical center from March to April 2020 and reported an 8% positivity rate with cough (51%), fever (41%), and myalgia (38%) being the most prevalent initial presenting symptoms and both anosmia (16%) and ageusia (15%) being less commonly reported among employees who tested positive for COVID-19. 

The mean time to RTW among COVID-19 positive patients in our sample also parallels previous findings of HCWs with mild to moderate illness. The retrospective cohort study of 1,698 HCWs with mild to moderate COVID-19 infection at a large academic medical center in the New York metropolitan area from March to June 2020 by Ganz-Lord et al. [11] found that employees who did not require hospitalization returned to work at a median of 15 days from symptom onset with dyspnea, fever, sore throat, and diarrhea being significantly associated with delayed RTW in contrast to our study where female gender, older age, and the presence of myalgia were noted to be predictive of longer illness duration. Of note, in their cohort, ageusia was also significantly associated with having a positive SARS-CoV-2 RT-PCR test and positive serology [11]. Moreover, Al Maskari et al. [12] performed a cross-sectional observational study exploring the characteristics of HCWs who tested positive for COVID-19 at a large tertiary hospital in Oman from March to July 2020; their reported RT-PCR positivity rate was 21.2%, and the most common clinical presentations among the 207 infected HCWs in their cohort were acute respiratory infection with fever (44%), acute respiratory infection without fever (36%), and headache (15%) with few reports of diarrhea (3%) and anosmia/ageusia (4%). It is worth noting that the prevalence of anosmia/ageusia in their sample was considerably lower than that of several other studies, including our study where 38.32% of COVID-19 positive patients endorsed a change in sense of smell and/or taste during their illness.

Tostmann et al. [13] reported findings of their cross-sectional survey of HCWs who underwent SARS-CoV-2 testing at their hospital in the Netherlands in March 2020 and found that test positivity was associated with non-respiratory symptoms (myalgia, ocular pain, general malaise, headache, extreme tiredness), including anosmia which was reported by 47% of positive HCWs in their cohort with an OR of 23.0. Moreover, Van Loon et al. [14] performed an observational study of HCWs who presented with mild symptoms of an acute respiratory illness at their large Belgian tertiary care center from March to April 2020 and reported a 49.9% positivity rate with cough, headache, myalgia, anosmia/ageusia, and fever being significantly more prevalent among employees who tested positive for SARS-CoV-2. Although Al Maskari et al. [12], Tostmann et al. [13], and Van Loon et al. [14] reported headache as a prevalent symptom among HCWs with confirmed COVID-19 infection, it is worth noting that we did not include this particular variable in our analysis as the presence or absence of this symptom was not routinely documented in patient charts.

While the aforementioned studies have explored COVID-19 symptomatology among the subpopulation of HCWs, there have also been similar studies examining the general population. For example, Dixon et al. [18] explored symptoms and symptom clusters associated with COVID-19 in a community-based population using statewide data in Indiana and reported that fever (OR = 5.34), anosmia (OR = 4.08), ageusia (OR = 2.38), and cough (OR = 2.86) were the individual symptoms most strongly associated with test positivity. In addition, the two symptom clusters most strongly associated with test positivity in their pooled cohorts were the triad of fever, ageusia, and anosmia and the triad of dyspnea, cough, and chest pain [18]. Morlock et al. [19] performed a cross-sectional nationwide survey of adults in the US between April and May 2020 and noted that, among adults who reported testing positive for COVID-19, the most commonly self-reported symptoms were dry cough, fever, and dyspnea. Moreover, predictors of test positivity included severe dry cough, new-onset anosmia/ageusia, acute respiratory issues, difficulty awakening from sleep, living with a symptomatic individual, recent international travel, and Black/African American race [19].

Given that high false-negative rates have been reported with SARS-CoV-2 RT-PCR assays, it is important to reiterate that our clinic adopted a testing strategy guided by clinical suspicion whereby patients would undergo repeat testing 24-48 hours after an initial negative test if the provider considered them to have a high likelihood of COVID-19 infection based on clinical judgment, including assessment of exposure risk, personal protective equipment worn by the source and employee, duration and distance of contact, and presenting viral symptoms. Arevalo-Rodriguez et al. [20] performed a systematic review and meta-analysis to estimate the proportion of SARS-CoV-2 false-negative results using RT-PCR assays obtained at the first healthcare encounter and found that up to 54% of COVID-19 patients may have an initial false-negative RT-PCR, which reinforces the need for repeat testing in patients with a negative initial test but a high clinical suspicion of COVID-19 infection. There have also been prior research aimed at elucidating factors associated with an initial false-negative SARS-CoV-2 RT-PCR test. For example, Lascarrou et al. [21] conducted a multicenter matched case-control study of patients with confirmed COVID-19 infection admitted to 11 hospitals in France and Belgium in which patients with a negative initial RT-PCR test were matched to patients with a positive initial RT-PCR test. The authors reported that two factors were independently associated with a lower risk of an initial false-negative test result (presence of headache and fatigue/malaise), whereas two factors related to marked inflammation were independently associated with a higher risk of an initial false-negative result (platelets > 207 x 10 mm-3 and c-reactive protein > 79.8 mg/L) [21].

Although our study was not designed to evaluate the economic impact of COVID-19-related absenteeism, our RTW data highlights the impact COVID-19 had on the healthcare workforce during a time when HCWs were more critical than ever. It was noted that COVID-19 positive employees returned to work, on average, 13 days from symptom onset. Moreover, although COVID-19 negative employees were discharged from our clinic and cleared for RTW sooner than their positive counterparts as one would expect, this still occurred at an average of seven days from symptom onset. This delay in RTW among COVID-19 negative HCWs was likely due to an institutional policy that required employees with any upper respiratory infection symptoms to RTW after their symptoms had improved despite testing negative for SARS-CoV-2. Moreover, there may have been delays in scheduling an appointment at our clinic, delays in undergoing testing, and delays in obtaining test results as there was a COVID-19 surge occurring in our geographic region during the study period, which was placing an increased burden on all local healthcare systems. On average, COVID-19 positive and negative HCWs were evaluated by an occupational medicine physician at 3.56 days and 3.29 days, respectively, from symptom onset during the study period. Therefore, our findings highlight that any evaluation of the economic impact of COVID-19-related absenteeism should include all employees who required self-isolation while awaiting testing and/or clinical evaluation in order to avoid underestimating the true economic impact of absenteeism related to COVID-19. 

Limitations

Although the present study included a large cohort of HCWs, it is not without its limitations. First, our study population only represents the proportion of HCWs who were evaluated at our institution’s OMC and, therefore, excludes HCWs who underwent testing alone without clinical evaluation and those who were evaluated by their primary care providers or other providers external to our clinic. In light of this, our test positivity rate does not reflect the true test positivity rate among all HCWs at our institution who were tested for SARS-CoV-2 during the study period-rather, it represents the test positivity rate in our cohort of patients who met the inclusion criteria. Selection bias could not be minimized as it is plausible that HCWs who were less symptomatic or asymptomatic following exposure to SARS-CoV-2 presented for COVID-19 testing and clinical evaluation at a lower rate than their more symptomatic counterparts. It is also important to note that our study includes almost three times as many women as men. Our disproportionate sample is likely attributed to the fact that the nursing workforce is predominantly female, and nursing staff is at increased risk of exposure due to the cumulative time spent at the patient’s bedside during work shifts. Moreover, there is also evidence indicating that women are more likely to utilize healthcare services than men [22]. Second, this cohort represents a relatively young and healthy population living within or in the vicinity of a designated “blue zone,” defined as a geographic region where individuals have been noted to live longer than average, who presented with only mild to moderate COVID-19 infection as severe cases were excluded by the nature of our sampling method. This, along with the healthy worker effect [23], cause us to assert that these results may not be generalizable to the general public.

Third, there was heterogeneity in testing as the PCR tests performed, testing sites, and laboratories were variable among this cohort of patients. In addition, the timing between symptom onset and PCR testing also varied as this was entirely dependent on when patients presented to our clinic and clinic appointment availability during a period of time in the pandemic when cases were surging in our geographic region, which may have resulted in delays in testing due to an overburdened system. Finally, this study design is subject to underestimation of symptoms as the symptom analysis is based entirely on self-reported symptoms documented in the EHR during the clinical encounter at variable time points in each patient’s disease course and, therefore, may not accurately reflect unreported symptoms that patients may have experienced prior to or after their telehealth visits with our providers.

## Conclusions

The COVID-19 pandemic significantly affected frontline HCWs. Having ample access to COVID-19 testing and data-driven CDC guidelines, our academic clinic was able to quickly identify, diagnose, isolate, and provide individualized treatment by judiciously applying CDC guidance to safely return employees back to work at our institution. Our findings confirm prior literature on clinical predictors of COVID-19 test positivity and add valuable insights regarding time to return to work following mild/moderate COVID-19 infection, including demographic and clinical predictors of delayed return to work among infected HCWs. Among HCWs with mild to moderate COVID-19 infection, discharge from our clinic occurred, on average, 13 days from symptom onset with female gender, older age, and myalgia being associated with delayed return to work.

Further research is warranted to evaluate overall symptomatic duration, potential long-term effects of COVID-19 infection, and the economic impact of COVID-19-related absenteeism. Nevertheless, our findings provide guidance for occupational medicine clinicians and employers as they continue to navigate the COVID-19 pandemic and reflect on how best to optimize their response to future communicable disease outbreaks.
